# The prognostic significance of tumour-stroma ratio in endometrial carcinoma

**DOI:** 10.1186/s12885-015-1981-7

**Published:** 2015-12-16

**Authors:** Hannah Panayiotou, Nicolas M. Orsi, Helene H. Thygesen, Alexander I. Wright, Matthew Winder, Richard Hutson, Michele Cummings

**Affiliations:** Section of Pathology and Tumour Biology, Leeds Institute of Cancer & Pathology, University of Leeds, St James’s University Hospital, Leeds, LS9 7TF UK; Netherlands Cancer Institute, Plesmanlaan 121, 1066 CX Amsterdam, Netherlands

**Keywords:** Endometrial cancer, Tumour-stroma ratio, Prognosis, Tumour microenvironment

## Abstract

**Background:**

High tumour stromal content has been found to predict adverse clinical outcome in a range of epithelial tumours. The aim of this study was to assess the prognostic significance of tumour-stroma ratio (TSR) in endometrial adenocarcinomas and investigate its relationship with other clinicopathological parameters.

**Methods:**

Clinicopathological and 5-year follow-up data were obtained for a retrospective series of endometrial adenocarcinoma patients (*n* = 400). TSR was measured using a morphometric approach (point counting) on digitised histologic hysterectomy specimens. Inter-observer agreement was determined using Cohen’s Kappa statistic. TSR cut-offs were optimised using log-rank functions and prognostic significance of TSR on overall survival (OS) and disease-free survival (DFS) were determined using Cox Proportional Hazards regression analysis and Kaplan-Meier curves generated. Associations of TSR with other clinicopathological parameters were determined using non-parametric tests followed by Holm-Bonferroni correction for multiple comparisons.

**Results:**

TSR as a continuous variable associated with worse OS (*P* = 0.034) in univariable Cox-regression analysis. Using the optimal cut-off TSR value of 1.3, TSR-high (i.e. low stroma) was associated with worse OS (HR = 2.51; 95 % CI = 1.22–5.12; *P* = 0.021) and DFS (HR = 2.19; 95 % CI = 1.15–4.17; *P* = 0.017) in univariable analysis. However, TSR did not have independent prognostic significance in multivariable analysis, when adjusted for known prognostic variables. A highly significant association was found between TSR and tumour grade (*P* < 0.001) and lymphovascular space invasion (*P* < 0.001), both of which had independent prognostic significance in this study population.

**Conclusions:**

Low tumour stromal content associates with both poor outcome and with other adverse prognostic indicators in endometrial cancer, although it is not independently prognostic. These findings contrast with studies on many - although not all - cancers and suggest that the biology of tumour-stroma interactions may differ amongst cancer types.

## Background

Endometrial cancer (EC) is the most prevalent gynaecological malignancy in the Western world and ranks as the ninth commonest cause of cancer-related mortality in women in the UK [1]. Moreover, the incidence of endometrial cancer in the UK has increased by 43 % in 15 years since 1993–1995, which has been accompanied by a 14 % increase in the number of EC-related deaths [2]. ECs are broadly categorised into types I and II on the basis of aetiology, histology and clinical behaviour [3, 4]. Type I (circa 80 % of cases) is represented by endometrioid endometrial carcinomas (EECs) which are typically oestrogen-dependent malignancies typically arising from a background of atypical hyperplasia. These tend to occur in younger, peri-menopausal women and generally have a more favourable outcome [4–7]. Most of the remaining 20 % of ECs are type II, high-grade, non-endometrioid endometrial cancers (NEECs) which are most commonly represented by serous and clear cell carcinomas. NEECs are thought to arise from a precursor intraepithelial carcinomatous lesion in a background of endometrial atrophy. These cancers tend to affect older, post-menopausal women, follow a more aggressive clinical course and have a much poorer prognosis [5]. However, this classification model is an over-simplification since many endometrial cancers are not categorised neatly according to this dichotomy. Indeed, poorly differentiated, high grade EECs are frequently grouped with the NEECs for the purpose of treatment due to their poorer outcome, although their prognosis in comparison to classical NEECs is debated [8–11]. Moreover, a large proportion of NEECs (circa 40 %) are of mixed subtype and can have endometrioid features [5]. Finally, the comparatively poor prognosis of low grade EECs arising in a background of atrophic endometrium present further difficulties for the Type I/II system [12]. Thus, there is a need to identify additional prognostic markers to achieve better patient stratification in the clinical management of endometrial cancer.

Malignant epithelial tumours are composed of carcinoma cells, together with stromal fibroblasts, immune effector cells, microvasculature and the extracellular matrix, which are collectively referred to as the tumour microenvironment. The dynamic interplay between cancer cells and stromal components within the tumour microenvironment contributes to malignant progression and metastasis [13]. As such, tumour-associated stroma has potential as both a target for novel therapies and utility in prognostication. A number of studies have identified tumour-stroma ratio (TSR) as having independent prognostic significance, where high stromal content has been shown to predict adverse outcome in a range of malignancies [14–27], although the prognostic significance of tumour stromal content in endometrial cancer remains to be determined. The purpose of this study was therefore to determine the prognostic significance of TSR and its association with other clinicopathological variables in a large series of surgically treated endometrial cancer patients, where TSR was assessed objectively using a digitised virtual scoring system.

## Methods

### Patients

This study received ethical approval from Leeds NRES committee (Ref: 05/Q1107/41). Patients gave their written informed consent for their tissue samples to be used in research. Clinicopathological and follow-up data were collected for a retrospective series of 400 women in the Yorkshire area (UK) diagnosed with endometrial adenocarcinoma between 2005 and 2007 who had undergone a hysterectomy at our tertiary referral centre (St James’s University Hospital). Median follow-up was 79.7 months (reverse Kaplan-Meier method). For both overall survival (OS) and disease-free survival (DFS), patients were censored at end of follow-up. OS was defined as time from diagnosis to death and DFS was defined as time from diagnosis to recurrence or death. Staging data were converted from the International Federation of Gynaecology and Obstetrics (FIGO) 1988 to the FIGO 2009 staging system [28] according to individual patients’ pathology reports.

### Morphometric assessment of tumour-stroma ratio

**Fig. 1:**
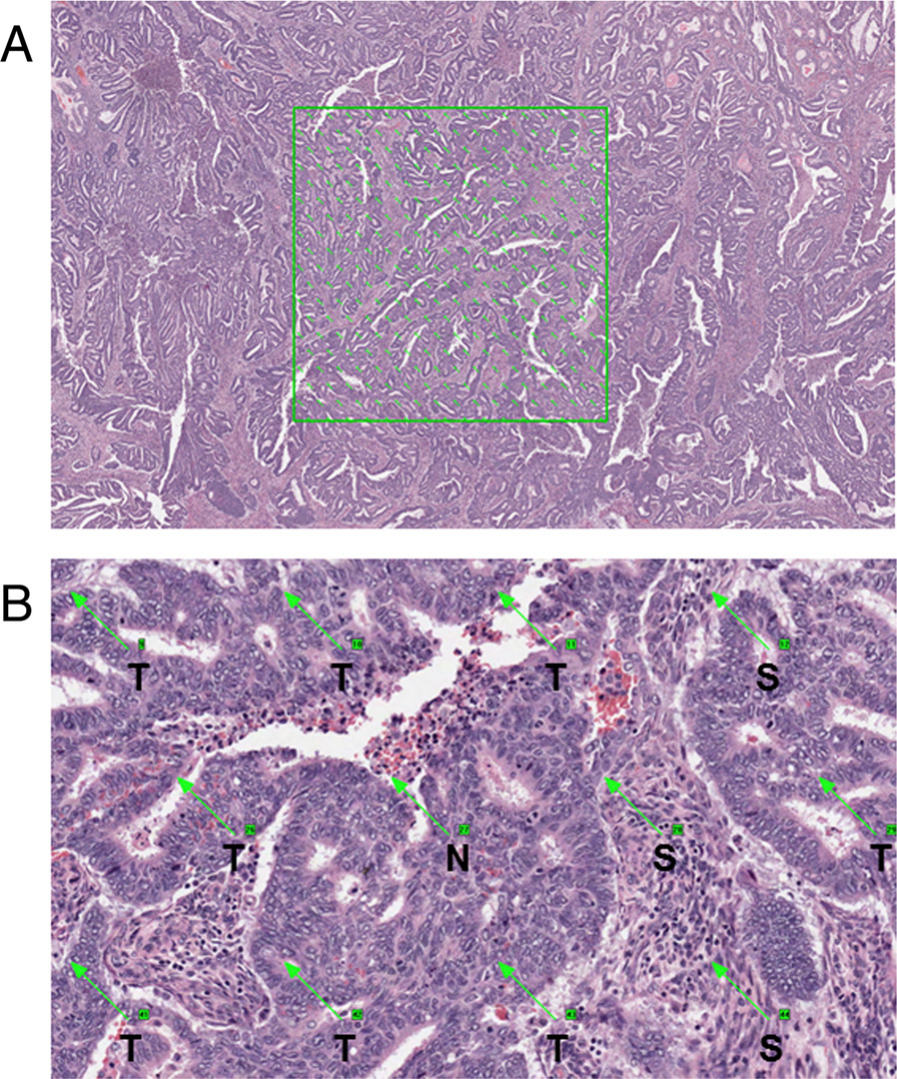
Morphometric assessment of tumour-stroma ratio. (**a**) Selection of a 9 mm^2^ area from a haematoxylin and eosin-stained representative section of endometrial cancer. A total of 300 points are randomly inserted into the selected area. (**b**) Annotation of individual points comprising tumour (T), stroma (S) and necrosis (N)

For each patient, 2 representative slides of 4 μm haematoxylin and eosin-stained tissue sections were selected and subjected to mark-up by a histopathologist (NMO). Areas selected for mark-up were from the superficial region, as in [17], in order to standardise sampling for all tumours since not all cases had significant myometrial invasion. Areas of overt necrosis and where tumour mass was poorly preserved were avoided. Each slide was scanned at 20× magnification using digital slide scanners (Aperio XT Aperio Technologies, Vista, CA, USA and hosted on the University of Leeds digital slide servers. An area of 9 mm^2^ (±0.25 mm^2^) was sampled from one slide for each patient using a digital slide viewer (ImageScope, Version 8.0, Aperio Technologies). In each instance, the slide that most accurately represented the tumour mass was used; in cases where more than one histological type was observed, multiple areas were marked up and sampled in order to obtain representative measures of tumour heterogeneity. Similarly, in large tumour masses where there could be variation in proportion of tumour and stroma, at least three 9 mm^2^ areas were defined. Virtual graticule software (RandomSpot) [29] was used to superimpose 300 (±15 %) systematic random points onto the selected area (Fig. 1); this number of measurement points has previously been optimised in other studies [17, 30]. The categories used, as devised by West and colleagues [17], were: uninformative (unclassifiable), tumour (viable cancer cell), stroma/fibrosis, necrosis, vessel, inflammation, tumour lumen (surrounded by tumour cells on all sides), mucus and smooth muscle. Retraction artefacts were classified in one of two ways: if the surrounding areas were the same histological category, i.e. retraction between two areas of stroma, then the retraction point was classified as that component. If the retraction artefact was between different histological categories i.e. between stroma and tumour, the retraction was classified as uninformative. Any tumour cells in areas of necrosis or lumenal debris were coded as necrosis. In areas of poor preservation where there was tumour breakdown, any debris or ‘white space’ were recorded as uninformative while clusters of viable cancer cells were recorded as tumour. Training for tumour scoring was provided by NMO and 20 cases were independently double-scored (HP and NMO). As inter-observer agreement between the two observers’ classifications was very high (κ = 0.94; see Statistical Analysis), the remainder of the cases were scored by one observer (HP).

Cases were marked up and scored with the observer blinded to the outcome data. Totals for each scoring category were generated and TSR was calculated by dividing the total tumour count over the total stroma count for each case.

### Statistical analysis

Inter-observer agreement on point classification (tumour, stroma etc.) was assessed using Cohen’s Kappa statistic. TSR was log-transformed prior to analysis to allow identical inferences for both tumour and stroma content to be made. Time-dependent survival analysis was used to optimise the TSR cut-off using *coxph* and *survfit* functions in the *R* package *Survival*, whereby the optimal cut-off gave the lowest log-rank *P* value. The prognostic significance of TSR, both as a continuous variable and using the TSR cut-off, on OS and DFS was determined using Cox Proportional Hazards regression analysis in *R* [31]. Kaplan-Meier analysis was conducted using IBM SPSS (version 21) and curves were visualised using Graphpad Prism (version 6). Associations of TSR with other clinicopathological variables were determined in SPSS using Mann–Whitney *U * tests or Kruskal-Wallis tests followed by Mann–Whitney*-U post-hoc* tests, as appropriate. Corrections for multiple comparisons were performed using Holm’s sequential Bonferroni method.

## Results

### Patient characteristics

Patient characteristics are summarised in Table 1. Median age at diagnosis was 66 years (range 28–95). In addition to total hysterectomy and bilateral salpingo-oophorectomy, 35 % of patients also underwent omental biopsy/omentectomy and 81 % had lymphadenectomy (pelvic/para-aortic). Following post-operative staging, 36 % of patients received adjuvant radiotherapy (brachytherapy and/or external beam radiotherapy) and 16 % of patients received adjuvant chemotherapy (paclitaxel and carboplatin combination therapy). None received neoadjuvant chemo/radiotherapy. The majority of patients (76 %) were diagnosed at early stage (I/II) and EEC was the predominant (76 %) histopathological subtype. There were 65 recurrences and 122 deaths during the follow-up period. The estimated cumulative 5-year survival for this patient cohort was 73.0 ± 0.02 % and 70.0 ± 0.02 % for OS and DFS, respectively.

**Table 1:** Summary of clinicopathological data for the patient cohort

Clinicopathological data	Median (range)
Age (years) at diagnosis	66 (28–95)
	*N* (%)
Histopathological subtype	
Endometrioid	302 (75.5)
Serous	34 (8.5)
Clear cell	11 (2.8)
Mixed	50 (12.5)
Undifferentiated	1 (0.25)
Mucinous	2 (0.5)
Surgical stage (FIGO 2009)	
I	262 (65.5)
II	39 (9.8)
III	75 (18.8)
IV	24 (6.0)
Grade	
1	149 (37.25)
2	106 (26.5)
3	145 (36.25)
Type of surgery	
Total abdominal hysterectomy	345 (86.3)
Laparoscopic assisted vaginal hysterectomy	55 (13.8)
Bilateral salpingo-oophorectomy	391 (97.8)
Lymphadenectomy	324 (81.0)
Omental biopsy	50 (12.5)
Omentectomy	89 (22.5)
Adjuvant therapy	
Radiotherapy alone	98 (24.5)
Chemotherapy alone	17 (4.25)
Radiotherapy + chemotherapy	45 (11.25)
No adjuvant treatment	240 (60)

*Abbreviation:* *FIGO* international federation of gynaecology and obstetrics

### Tumour-stroma ratio and cut-off determination

**Fig. 2:**
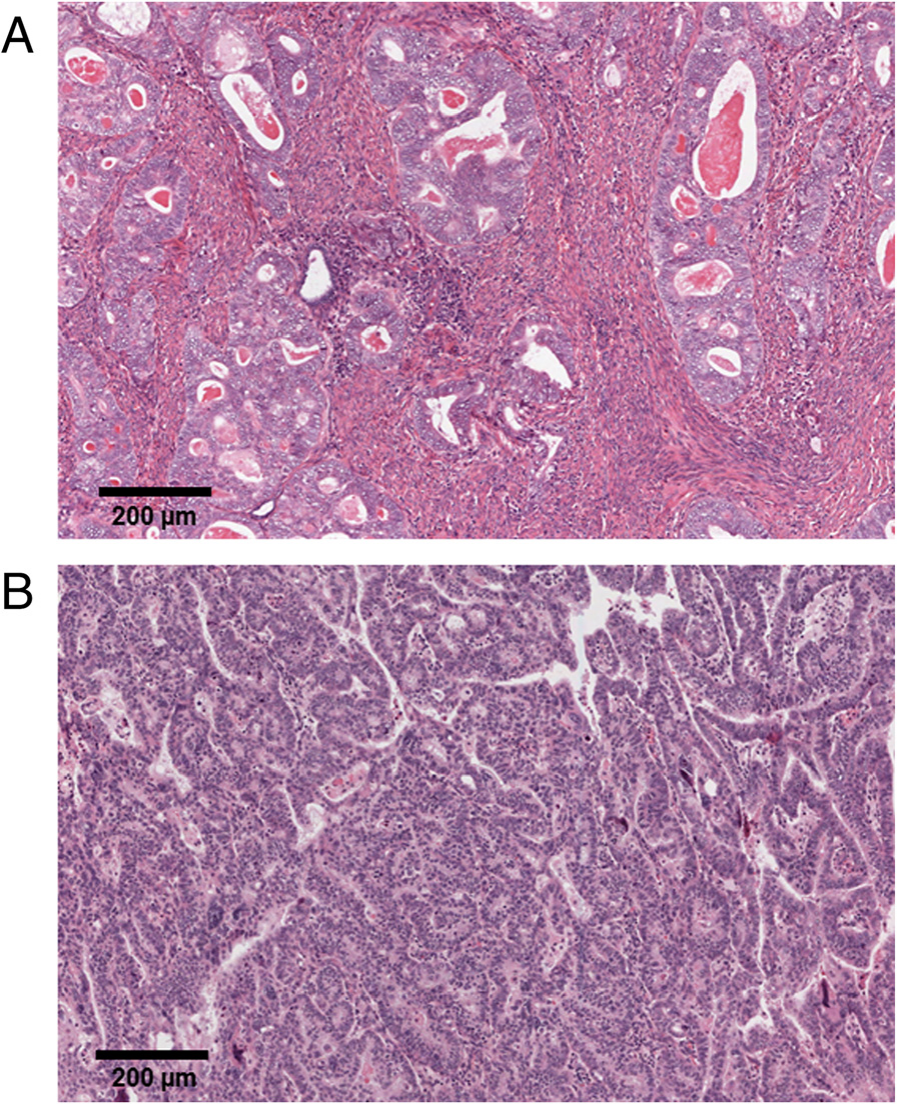
Representative examples of TSR-low and TSR-high endometrial cancer specimens. Haematoxylin and eosin-stained sections of (**a**) TSR-low and (**b**) TSR-high EEC cases

Including all histological types, the median percentage fraction of tumour was 66.0 % (range 12.7–92.2 %) whilst the median percentage fraction of stroma was 20.1 % (range 2.0–81.2 %). The median TSR was 3.3 (range 0.16–45.20). TSR cut-off optimisation identified a TSR cut-off of 1.3 for OS which, in an idealised sample with only tumour and stroma scores, would correspond to a tumour-stroma ratio of 56.5 %:43.5 %. Representative images of TSR low and TSR high tumours are depicted in Fig. 2.

### Increased TSR associates with adverse prognosis in univariable analysis

**Fig. 3:**
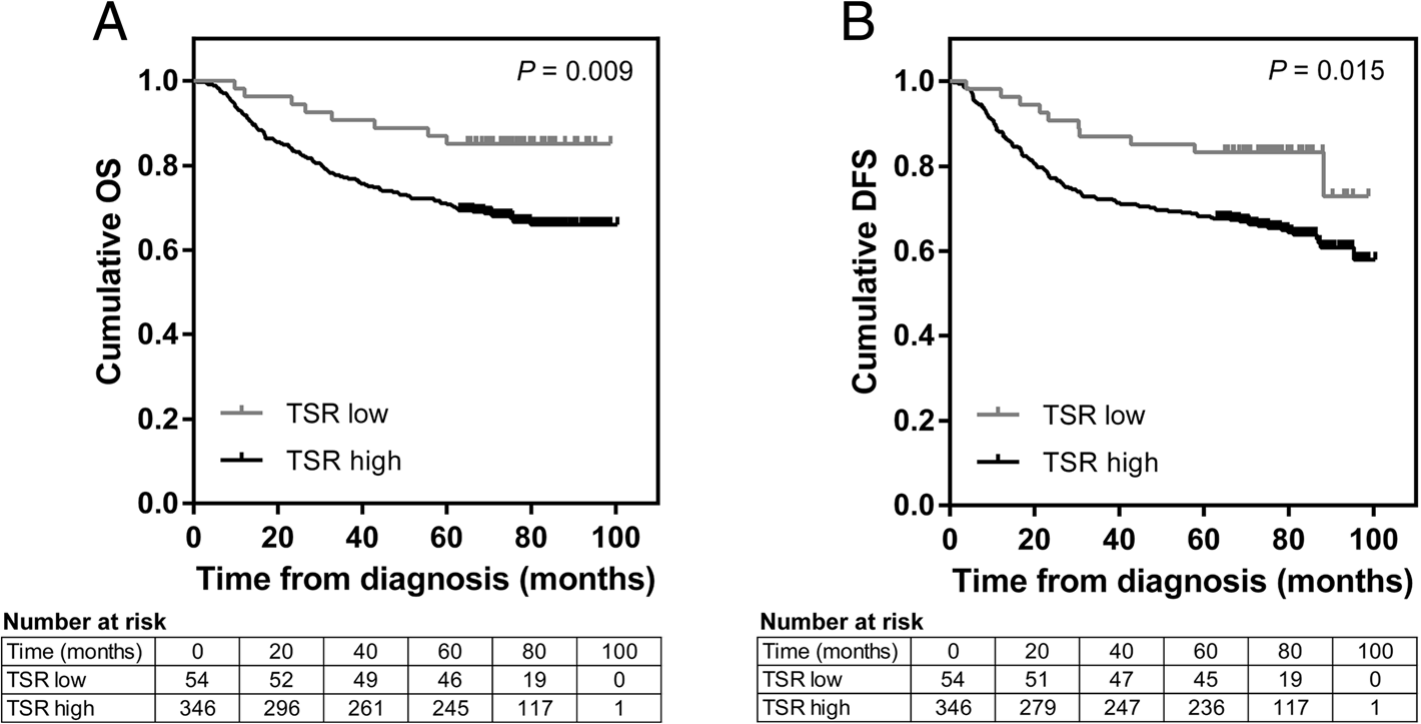
Kaplan-Meier survival curves of patients dichotomised according to the optimised TSR cut-off. Kaplan–Meier overall (**a**) and disease-free (**b**) survival curves plus log-rank *P*-values of patients dichotomised according to a TSR cut-off of 1.3. Numbers at risk for each group are tabulated below each graph. Abbreviation: TSR = tumour-stroma ratio

Prognostic parameters for univariable analysis included age, FIGO 2009 stage, grade, and the presence of lymphovascular space invasion, a known independent prognostic indicator for endometrial cancer [32]. Depth of myometrial invasion, cervical involvement and lymph node status form part of the FIGO staging system and, as such, were not included as independent variables in the analysis. Univariable Cox proportional hazards analysis of logTSR as a continuous variable showed that increased TSR was significantly associated with worse OS (*P* = 0.032) and showed a trend towards associating with poorer DFS (P = 0.058) (Table 2). Kaplan-Meier analysis of patients stratified according to the optimised TSR cut-off of 1.3 revealed that high TSR (stroma-low) tumours were significantly associated with worse OS (*P* = 0.009) and DFS (*P* = 0.015) (Fig. 3). Estimated five-year cumulative OS and DFS rates were 85 % and 83 %, respectively, for the TSR-low (stroma-high) group versus 71 % and 68 %, respectively, for the TSR-low group. Univariable Cox regression analysis confirmed that TSR-high tumours (stroma-low) were associated both with significantly worse OS and DFS (Table 2). However, TSR did not have independent prognostic significance in multivariable analysis when adjustments were made for age, stage, grade, and lymphovascular invasion (Table 3). Significant independent prognostic variables for the study cohort were age, stage, grade and lymphovascular invasion for OS, and age, stage and lymphovascular invasion for DFS (Table 3).

**Table 2:** Univariable survival analysis of TSR and other prognostic factors

	Overall survival		Disease-free survival	
Factor	HR (95 % CI)	*P*	HR (95 % CI)	*P*
LogTSR (continuous)	1.75 (1.04–2.94)	0.034	1.61 (0.98–2.64)	0.058
TSR ( ≥1.30 *vs*. <1.30)	2.51 (1.22–5.14)	0.012	2.18 (1.15–4.16)	0.017
Age (continuous)	1.07 (1.05–1.09)	<0.001	1.06 (1.05–1.08)	<0.001
Stage (FIGO 2009)				
I	Referent	Referent	Referent	Referent
II	1.83 (1.00–3.36)	0.051	1.69 (0.94–3.01)	0.078
III	3.21 (2.10–4.90)	<0.001	2.93 (1.96–4.39)	<0.001
IV	11.44 (6.72–19.32)	<0.001	9.15 (5.52–15.15)	<0.001
Grade				
1	Referent	Referent	Referent	Referent
2	1.61 (0.95–2.75)	0.080	1.53 (0.93–2.49)	0.092
3	3.49 (2.22–5.49)	<0.001	2.95 (1.94–4.48)	<0.001
Lymphovascular invasion (yes *vs*. no)	3.00 (2.04–4.42)	<0.001	2.81 (1.95–4.04)	<0.001

Univariable Cox proportional hazards regression for overall and disease-free survival. TSR was analysed both as a continuous variable (logTSR) and dichotomised according to the optimised cut-off

*Abbreviations:* *CI* confidence interval, *FIGO* international federation of gynaecology and obstetrics, *HR* hazard ratio, *TSR* tumour-stroma ratio

**Table 3:** Multivariable survival analysis of TSR and other prognostic factors

	Overall survival		Disease-free survival	
Factor	HR (95 % CI)	*P*	HR (95 % CI)	*P*
TSR ( ≥1.30 *vs*. <1.30)	1.18 (0.56–2.47)	0.667	1.12 (0.57–2.18)	0.740
Age (continuous)	1.07 (1.05–1.09)	<0.001	1.06 (1.04–1.08)	<0.001
Stage (FIGO 2009)				
I	Referent	Referent	Referent	Referent
II	1.12 (0.60–2.09)	0.731	1.10 (0.60–2.00)	0.603
III	1.69 (1.07–2.67)	0.024	1.64 (1.06–2.54)	0.028
IV	8.38 (4.75–14.74)	<0.001	6.77 (3.92–11.70)	<0.001
Grade				
1	Referent	Referent	Referent	Referent
2	1.06 (0.61–1.86)	0.835	0.95 (0.57–1.61)	0.857
3	1.96 (1.20–3.21)	0.007	1.61 (1.02–2.55)	0.042
Lymphovascular invasion (yes *vs*. no)	1.94 (1.26–2.90)	0.002	1.95 (1.30–2.94)	0.001

Multivariable Cox proportional hazards regression for overall and disease free survival

*Abbreviations:* *CI* confidence interval, *FIGO* International Federation of Gynaecology and Obstetrics, *HR* hazard ratio, *TSR* tumour-stroma ratio

### TSR associates strongly with tumour grade and the presence of lymphovascular invasion

Potential associations of TSR with other clinicopathological variables were also investigated. After correction for multiple comparisons, TSR was significantly higher in grade 3 vs. grade 1 carcinomas (P < 0.001) as well as in tumours with lymphovascular invasion (*P* < 0.001). TSR was also higher in the tumours of patients aged ≥75 years compared with patients aged <55 years, although this association was weaker (*P* = 0.019). TSR was not significantly associated with any other clinicopathological variable, including stage, histopathological subtype, depth of myometrial invasion, lymph node status or cervical involvement (Table 4). Thus, although high TSR associates with certain adverse prognostic features in EC, it does not provide additional prognostic information independent of these features.

**Table 4:** Association of TSR with other clinicopathological factors

Factor	*N* (%)	TSR, median (IQR)	*P*
All patients	400 (100)	3.3 (2.0–5.3)	
Age			
<55	56 (14)	3.0 (1.8–4.0)^a^	0.019
55–64	125 (31)	3.4 (2.0–5.3)^a,b^	
65–74	134 (34)	3.0 (1.9–5.4)^a,b^	
≥75	85 (21)	4.7 (2.5–6.0)^b^	
Stage (FIGO 2009)			
I	230 (58)	3.0 (1.8–4.9)	0.192
II	71 (18)	4.0 (2.5–6.6)	
III	75 (19)	3.6 (2.1–5.3)	
IV	24 (6)	4.8 (2.6–6.2)	
Grade			
1	149 (37)	2.8 (1.7–4.8)^a^	<0.001
2	106 (27)	3.2 (1.9–4.9)^a,b^	
3	145 (36)	4.1 (2.3–6.0)^b^	
Histology			
Endometrioid (EEC)	302 (75.5)	3.2 (2.0–5.1)	1.000
Non–EEC	48 (12)	3.9 (2.2–6.6)	
Mixed EEC/non-EEC	50 (12.5)	3.3 (1.9–5.4)	
Depth of myometrial invasion			
Inner half	210 (52.5)	3.1 (1.7–5.6)	1.000
Outer half	190 (47.5)	3.6 (2.2–5.0)	
Cervical involvement			
No	261 (65)	3.0 (1.7–5.0)	0.108
Yes	128 (32)	3.7 (2.3–5.4)	
Missing data	11 (3)	-	
Lymph nodes positive			
No	274 (68.5)	3.3 (2.0–5.3)	1.000
Yes	50 (12.5)	3.8 (2.2–5.8)	
No lymphadenectomy	76 (19)	-	
Lymphovascular invasion			
No	203 (51)	2.9 (1.6–4.8)	<0.001
Yes	193 (48)	3.9 (2.2–5.8)	
Missing data	4 (1)	-	
Adjuvant treatment			
No	240 (60)	3.2 (1.9–5.2)	1.000
Yes	160 (40)	3.6 (2.1–5.4)	

Data were analysed by Mann–Whitney *U* tests or Kruskal-Wallace tests, as appropriate. *P*-values following correction for multiple comparisons (Holm’s sequential Bonferroni method) are indicated. ^a,b^Depict significant differences between categories following post-hoc Mann–Whitney *U* tests

*Abbreviations:* *EEC* endometrioid endometrial carcinoma, *FIGO* international federation of gynaecology and obstetrics, *IQR* interquartile range

## Discussion

The stromal component of epithelial tumours is an area of intense research, given the importance of the tumour microenvironment in cancer progression [13, 33]. In this respect, TSR could be viewed as an indirect measure of the stromal contribution to malignant progression, as suggested by studies showing an association between high tumour stromal content and adverse clinical outcome in colorectal [14, 17, 22, 25], oesophageal [15, 20], gastric [34] nasopharyngeal [26] breast (particularly triple negative) [18, 19, 21, 23] hepatocellular [27], prostate [35] ovarian [16] and cervical [24] cancers. These results contrast with the findings of the current study, which demonstrate that high tumour stromal content (i.e. low TSR) associates with better prognosis in endometrial cancer, both as a continuous variable and when applying an optimised TSR cut-off. Moreover, the present data identify highly significant positive associations between TSR and adverse prognostic features for EC, namely, grade 3 carcinomas and the presence of lymphovascular invasion. These observations may account for the lack of independent prognostic significance of TSR in EC, but also underscore the association of high stromal content with good prognosis in this tumour type. The observation that high stromal content is not a universal adverse prognostic feature is corroborated by recently published studies demonstrating low TSR to be associated with favourable outcome in both oestrogen receptor positive breast cancer [30] and pancreatic cancer [36]. The majority of studies investigating the prognostic significance of TSR have employed visual estimation of stromal content, unlike the systematic scoring method applied herein, which could account for differences in TSR cut-off selection and subsequent outcome prediction. However, in colorectal cancer, where both methods have been applied in independent studies, there is agreement between the systematic scoring method [17] and conventional visual estimation [14, 22, 25], both in terms of TSR cut-off estimation and prognostic significance.

Reactive stromal formation is a recognised feature of the inflammatory tumour microenvironment and is characterised by the presence of cancer associated fibroblasts (CAFs), which have been shown to be active players in tumour progression and metastasis through their bidirectional interactions with cancer cells (as well as other cells within the tumour microenvironment) via cytokine/growth factor mediated signalling and extracellular matrix remodelling [37–39]. However, this paradigm may not necessarily apply to all cancer types. Indeed, recent studies demonstrating a tumour-suppressive role for CAFs and fibrosis in pancreatic cancer [40, 41], together with the association of high tumour stromal content with good prognosis in pancreatic cancer reported by Bever and colleagues [36], suggest that fundamental cancer type-specific differences in tumour-stroma interactions may exist. Although isolated endometrial CAFs have been found to promote cancer cell growth [42] or migration [43] *in vitro*, a recent study investigating stromal mRNA and protein expression signatures in EEC found that the macrophage response signature rather than the activated stromal signature associated with adverse prognostic features [44]. As yet, the role of the tumour microenvironment in EC progression is less well studied than in other common cancers. Clearly, better characterisation of the EC tumour microenvironment, including the involvement of the immune effector cell infiltrate, will be necessary for more accurate prognostication and development of stromal-targeted therapeutic strategies.

The advantages of this study are its large cohort size and comparatively long follow-up period. Another advantage is the use of a digitised scoring method, which provides a framework for the objective measurement of TSR. One potential limitation is the heterogeneity of EC subtypes included in the study. However, TSR was not found to differ significantly between endometrioid/non-endometrioid/mixed histology subtypes, thus justifying such an inclusive approach.

## Conclusions

In summary, this study shows that low tumour stromal content associates both with poor outcome and adverse prognostic features endometrial cancer, although it is not independently prognostic. These findings are consistent with the idea that the biology of tumour-stroma interactions and their prognostic influence are not universal amongst epithelial tumours.

## Abbreviations

CAF: cancer-associated fibroblast
CI: confidence interval
DFS: disease-free survival
EC: endometrial cancer
EEC: endometrioid endometrial cancer
FIGO: international federation of gynaecology and obstetrics
HR: hazard ratio
NEEC: non-endometrioid endometrial cancer
OS: overall survival
TSR: tumour-stroma ratio
